# The potential of live biotherapeutic products in allergic disease: current findings and future directions

**DOI:** 10.3389/frmbi.2024.1418633

**Published:** 2024-07-09

**Authors:** Isabel Tarrant, B. Brett Finlay

**Affiliations:** ^1^ Department of Microbiology and Immunology, University of British Columbia, Vancouver, BC, Canada; ^2^ Michael Smith Laboratories, University of British Columbia, Vancouver, BC, Canada; ^3^ Department of Biochemistry, University of British Columbia, Vancouver, BC, Canada

**Keywords:** allergic disease, asthma, live biotherapeutic products, microbiome, probiotics, early life, childhood allergy

## Abstract

With the global prevalence of allergic disease continuing to rise at an alarming rate, the need for effective and safe therapeutics is paramount. Given the critical role of the early-life microbiota on immune development, emerging research suggests the potential use of live biotherapeutic products (LBP) for the prevention and treatment of childhood allergy. However, findings are limited and inconsistent. Therefore, the present review critically evaluates the current animal and human data on the therapeutic value of LBPs in allergy, the underlying immunological mechanisms by which LBPs may mediate allergy susceptibility, limitations of the current research that need to be addressed, and future research directions. Accordingly, LBPs may protect against allergic disease through several immunological and physiological mechanisms during early-life, including regulation of Th1/Th2 balance, SCFA-induced activation of GPR41/43 and HDAC inhibition, and maturation of epithelial barrier integrity. Taken together, current findings indicate powerful immunomodulatory properties of LBPs on allergic immune response, with LBPs offering exciting potential as a novel therapeutic tool for childhood allergy. However, the efficacy of LBPs in allergy is complex and influenced by many population and methodological factors, resulting in varied therapeutic benefits. While research thus far has focused on traditional probiotic strains, greater investigation into microbial consortiums selected from the microbiota of non-allergic infants may provide greater promise as a therapeutic tool for allergic disease. Further investigation, particularly into long-term efficacy, strain-specific effects, optimal supplementation regimes, and use of multi-strain consortiums, is necessary before findings can be translated into clinical applications to tackle childhood allergic disease.

## Introduction

Atopy, an exaggerated IgE response to an allergen, affects 1 in 5 individuals worldwide (Bantz et al., 2014), with prevalence rapidly rising across the globe (Gutowska-Ślesik et al., 2023). Particularly in developing countries, rates of childhood allergy, including asthma, allergic rhinitis, atopic dermatitis and food allergy, collectively known as the atopic march, have seen drastic increases (Wong et al., 2013; Eghtesad, 2020; Edwards-Salmon et al., 2022), with 30% of individuals now estimated to be affected by atopic disease (Sánchez-Borges et al., 2018). Asthma, for instance, is the most common chronic childhood disease, with prevalence expected to affect 400 million people globally by 2025 (Masoli et al., 2004). Accordingly, the economic burden of allergic disease is considerable, with asthma alone costing the US $50 billion per year (Hester et al., 2016), and pediatric food allergies costing $25 billion (Gupta et al., 2013). Allergic disease has significant impacts on quality of life, with asthma accounting for 13.8 million missed school days in the US, and ∼250,000 premature deaths globally per year (Pawankar, 2014). Unfortunately current treatment options are costly, often ineffective long-term, and are associated with many adverse effects, such as corticosteroid dependency (Lefebvre et al., 2015; Volmer et al., 2018). Moreover, little attention has been paid to the prevention of allergic disease, with current interventions focused predominately on treatment. Thus, atopic disease represents a major global health and economic burden, and the need for safe and effective therapeutics, particularly prophylactics, is paramount.

Live biotherapeutic products (LBPs), defined as the use of live microorganisms including bacteria, viruses and fungi for the prevention or treatment of disease (Cordaillat-Simmons et al., 2020), may represent one such potential therapeutic tool for childhood allergy. In recent years, emerging research has demonstrated a link between the early-life intestinal microbiota and risk of allergic disease. Significant differences in microbiome composition are repeatedly reported between infants with allergic disease compared to healthy counterparts (Kourosh et al., 2018; Boutin et al., 2020; Lee-Sarwar et al., 2023). Moreover, perturbations to microbiome colonization in early-life, i.e., via antibiotic use, are associated with increased allergy risk in childhood (Ni et al., 2019; Patrick et al., 2020). Accordingly, germ-free mice show enhanced susceptibility to allergic disease, compared to those with an established microbiota (Rodriguez et al., 2011), highlighting the important role of the intestinal microbiome in allergy development. The clear link between early-life microbiome development and allergy susceptibility has led to the exciting potential of microbiota-targeted interventions, including LBPs, for the prevention and treatment of atopic disease. However, research is still in its infancy, with many issues, including the identification of optimal bacterial strains, yet to be addressed before clinical recommendations on the therapeutic use of LBPs for childhood allergy can be made.

Consequently, this review will critically evaluate the role of the early-life intestinal microbiome in atopic disease and the current available research regarding the therapeutic potential of LBPs in allergy. Moreover, the underlying mechanisms by which LBPs may modulate allergic immune response, current challenges of LBP application in allergy and future research directions, will be assessed.

At this point, it is important to note the distinction between probiotics and LBPs, with probiotics defined as live microorganisms that confer benefit to host health, and LBPs as live microorganisms used for the prevention or treatment of disease (Cordaillat-Simmons et al., 2020). Accordingly, this distinction is not based on the content of the product, but it’s regulatory status as a therapeutic for disease. Therefore, although some probiotic bacteria may fall under the category of LBPs due to their therapeutic effects on disease, not all probiotics can be classified as LBPs.

In addition, it should be noted that aside from the intestinal microbiota, the development of both the oral and respiratory microbiomes during early-life also influences immune function and allergy susceptibility (Teo et al., 2018; Arweiler et al., 2021; Cui et al., 2021; Ho et al., 2021). However, given the emerging data on the use of intestinal-targeted LBPs in allergy, this review will focus predominately on the role of the gut microbiome in allergy, and specifically, the potential of LBPs for the prevention and treatment of allergic disease.

## Early-life intestinal microbiome shapes immune development and allergy susceptibility

The early-life gut microbiota profoundly impacts immune development, having long-lasting effects on disease outcomes later in life (Gensollen et al., 2016; Iyer and Blumberg, 2018). It is well established that the first ∼1000 days of life are a critical window of opportunity in which the maturing gut microbiota drastically shapes immune programming, and that perturbations to the microbiota during this critical window can have lasting impacts on immune function and subsequent disease risk, including allergy susceptibility (Cukrowska, 2018; Romano-Keeler and Sun, 2022). Accordingly, disruptions to microbiome maturation during the early-life critical window, through factors such as caesarean-delivery (Huang et al., 2015), method of feeding (Hu et al., 2021), prenatal (Cait et al., 2022) and infant antibiotic use (Ni et al., 2019) and pet exposure (Hesselmar et al., 2018), influence immune development and subsequent risk of atopy. For instance, both early-life antibiotic exposure (Lu et al., 2023) and caesarean-delivery (Liang et al., 2023) are associated with increased risk of asthma in childhood, reflecting the integral relationship between the gut microbiota and immune education.

Immune development begins in-utero, and despite the longstanding belief that the womb is sterile, recent findings suggest microbiota colonization commences in-utero (Stinson et al., 2019; Mishra et al., 2021) and influences fetal immune programming (Mishra et al., 2021). Neonatal immune regulation is initiated prenatally, in part driven by transplacental transfer of microbiota-derived metabolic signatures, such as short-chain fatty acids (SCFAs), which are able to cross the placenta and influence fetal T cell development (Kimura et al., 2020; Husso et al., 2023). For instance, low serum acetate levels during pregnancy are associated with impaired Treg production in neonates (Hu et al., 2019). In accordance, prebiotic supplementation increased fecal acetate concentration of pregnant dams, which passed through the placenta and was detected in amniotic fluid, and elevated Treg cell production in the fetus, thus having pro-tolerogenic effects (Brosseau et al., 2021). Moreover, several bacterial strains, including *Staphylococcus* and *Lactobacillus* isolated from second-trimester fetal tissues were found to induce *in-vitro* activation of memory T cells in the fetal mesenteric lymph node, supporting the role of prenatal microbial exposure on fetal immune programming, particularly T cell development (Mishra et al., 2021). Notably, Th1/Th2 balance is a critical component of allergic inflammation; Type 2 allergies involve over-activation of Th2 cells in response to an antigen, triggering the production of type 2 cytokines, namely IL-4, IL-5 and IL-13, signaling an inflammatory cascade resulting in antigen specific-IgE production, mast cell activation and eosinophil degranulation, ultimately leading to the manifestation of allergy symptoms (Caminati et al., 2018; Akdis et al., 2020).During pregnancy, the fetal immune system presents a Th2-dominant phenotype, and the Th1 response is suppressed, resulting in Th2 polarization (Lee C-L. et al., 2011). However, microbial exposures during the pre- and post-natal period, promote a gradual shift from Th2 dominance to Th1/Th2 homeostasis (Qian et al., 2017), fostering immunotolerance to antigens. As such, maternal abundance of *Prevotella*, a SCFA-producing genus, during pregnancy is associated with reduced risk of food allergy in infants (Vuillermin et al., 2020), emphasizing the impact of the maternal prenatal microbiome on infant immune programming. Dysregulation of this Th2-Th1 shift in early-life, due to a variety of environmental and genetic factors, can result in excessive Th2 activation and improper maturation of Th1/Th2 balance (Martino et al., 2018; Krusche et al., 2020; Zhang et al., 2021) thus leading to increased allergy susceptibility. For instance, maternal antibiotic exposure during pregnancy is associated with an increased risk of both asthma and atopic dermatitis (AD) in offspring (Zhong et al., 2021), highlighting the influential role of the maternal microbiota on fetal immune development.

**Figure 1:**
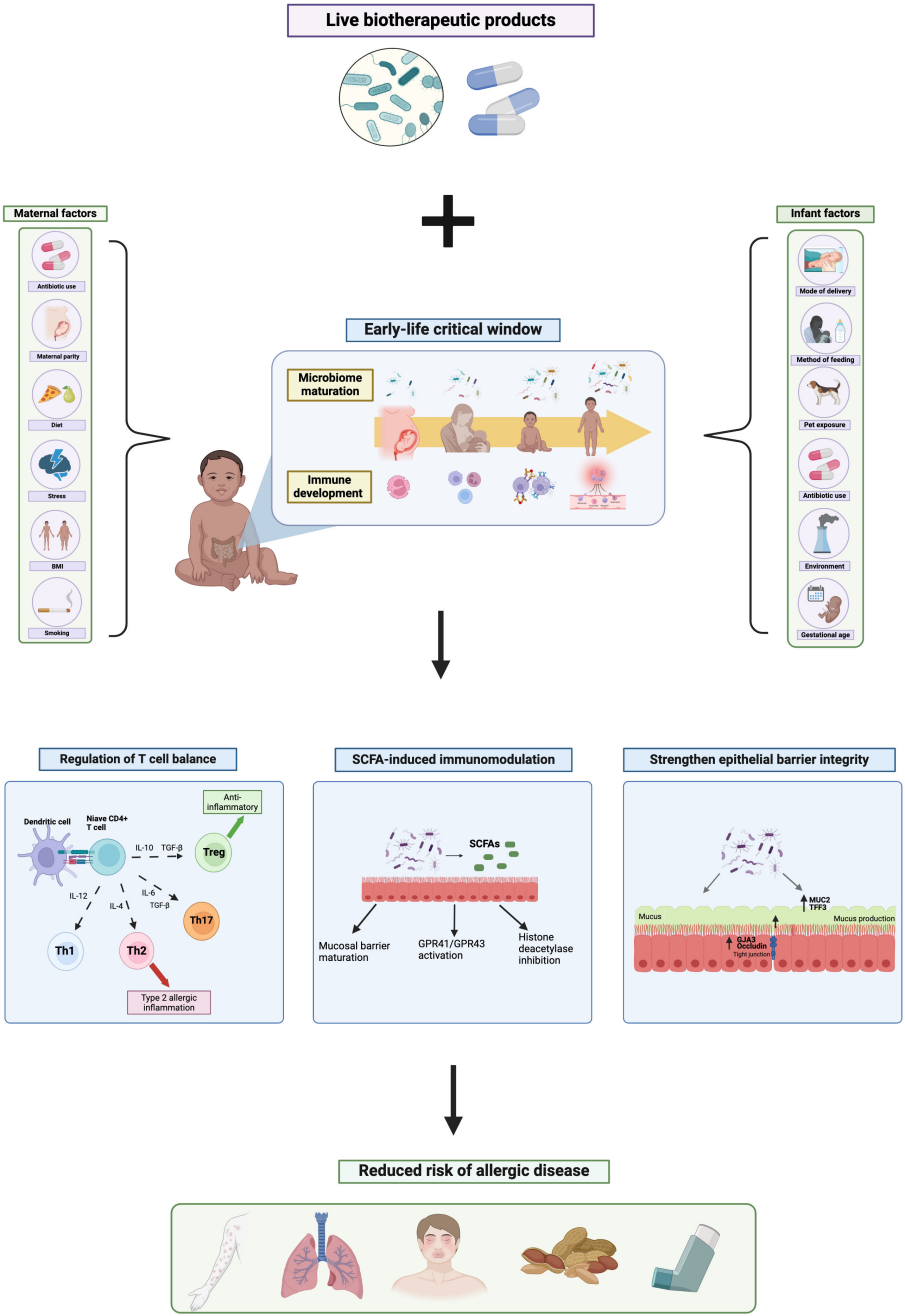
The mechanistic role of live biotherapeutic products on the early-life gut microbiome and risk of allergic disease. Created with BioRender.com.

Microbiota-initiated immune programming continues into the post-natal period, shaped by many early-life microbial exposures such as mode of delivery, antibiotic use, pet exposure, urban vs. rural habitation, and method of feeding, as seen in **Figure 1** (Kumbhare et al., 2019; Donald and Finlay, 2023). Breast milk, for instance, contains a multitude of bioactive components, including commensal bacteria, antibodies, human milk oligosaccharides (HMOs) and immune cells, which promote maturation of both the infant immune system and microbiota, and consequently help protect against immune-mediated disorders including allergy (Lokossou et al., 2022). Breast milk-transmitted maternal IgA is found to bind to intestinal bacteria (Sterlin et al., 2019) which not only helps prevent infection (Guo et al., 2021), but also dampens antigen-stimulated immune response by suppressing T helper cell activation (Koch et al., 2016), thus contributing to early-life immune regulation via microbiota-dependent mechanisms. Accordingly, sIgA treatment ameliorates allergic inflammation and promotes oral tolerance in a mouse model of food allergy (Kizu et al., 2015). Similarly, HMOs found in breast milk are shown to prevent the development of asthma in a murine model (Bozorgmehr et al., 2023), which is thought to reflect their prebiotic effect on the infant microbiota, promoting increased SCFA production, as well as supporting mucus production and epithelial barrier integrity (Tarrant and Finlay, 2023). In addition, a study comparing breastfed and formula fed infants demonstrated that breast milk promotes immunotolerance to antigens by increasing the production of Treg cells and suppressing T helper cell differentiation and cytokine production (Wood et al., 2021). Notably, here breastfed infants demonstrated significantly increased fecal abundance of SCFA-producing bacteria, compared to formula-fed, indicating the microbiota-dependent mechanism of breastfeeding in allergy protection.

Moreover, environmental exposures during both the pre- and post-natal period have significant impacts on infant microbiota development and thus subsequent allergy risk (Sbihi et al., 2019). For instance, growing up on a farm is found to be protective against asthma, atopic dermatitis and allergic rhinitis (Feng et al., 2016; Botha et al., 2019; Depner et al., 2020) due to increased exposure to farm-associated microbes, such as *Lachnospiraceae* and *Ruminococcaceae* (Kirjavainen et al., 2019; Depner et al., 2020), in early life resulting in enhanced microbial diversity and maturation, thus influencing immune-programming and allergy outcomes. Conversely, urban habitation is repeatedly associated with increased risk of allergic disease (Botha et al., 2019; Lehtimäki et al., 2021) with urbanized infants showing significant differences in intestinal microbiota composition to that of rural infants (Lehtimäki et al., 2021). Notably, this urbanized-microbiota signature is correlated with elevated inflammatory marker concentrations by 1 month of age, including CXCL8, CCL2 and CCL17, decreased levels of anti-inflammatory cytokine IL-10, and increased risk of asthma, atopic dermatitis and allergic sensitization throughout childhood (Lehtimäki et al., 2021). Specifically, infants exposed to pets or farm environments in early life show elevated fecal abundance of SCFA-producing bacteria (Gio-Batta et al., 2020; Yang et al., 2022). Notably, SCFAs have powerful anti-inflammatory and allergy-protective effects, through their ability to regulate T helper cell balance and support epithelial barrier maturation (Ratajczak et al., 2019), as discussed later in this review.

Consequently, maturation of the infant intestinal microbiota, influenced by a variety of environmental exposures commencing in the womb and continuing throughout the early-life critical window, drives immune programming, thus influencing subsequent immunotolerance and risk of allergic disease.

Given our growing understanding of the profound impact of the infant microbiota on immune programming and allergy susceptibility, determining the therapeutic potential of microbiota-targeted interventions, such as LBPs, for the prevention and treatment of allergic disease is of significant value.

## Mechanisms of LBPs in allergy

### Regulation of Th1/Th2 balance

One mechanism by which LBPs may influence childhood allergy susceptibility, is by regulation of Th1/Th2 cell homeostasis. As mentioned above, T helper cell imbalance is a critical component of allergic immune response, with allergic inflammation ascribed to enhanced activation of Th2 cells in response to an allergen, triggering the production of type 2 cytokines and IgE, eosinophil and mast cell degranulation, and ultimately allergy symptoms. Regulatory T cells on the other hand, exhibit anti-inflammatory effects by suppressing over-activation of Th2 cells, maintaining Th1/Th2 homeostasis, and promoting immunotolerance to allergens. Probiotic bacteria are able to bind to toll-like-receptors (TLRs), including TLR2, TLR4 and TLR6, on dendritic cells (Ren 2016; Bermudez-Brito) and stimulate dendritic cell maturation towards IL-10 production, resulting in Treg differentiation and thus immune regulation (Hisbergues et al., 2007). For instance, *Lactobacillus plantarum* treatment induced the production of anti-inflammatory IL-10 and IL-12 from allergen-stimulated bone marrow-derived dendritic cells (BMDCs) isolated from asthmatic mice, via activation of TLR4 and TLR2 signalling pathways (Adam et al., 2010). Similarly, in another study, not only did *Escherichia coli* Nissle (EcN) supplementation prevent allergic inflammation in a mouse model of asthma, when treated to murine BMDCs, EcN was able to activate dendritic cell maturation towards IL-10 production, up-regulation expression of DC maturation factors CD40, CD80 and CD86, and suppress type 2 cytokine production in a TLR4-dependent manner (Adam et al., 2010). These findings translate *in-vivo*; *Bifidobacterium*, *Clostridia*, and *Lactobacillus* supplementation suppresses Th2 skewing and stimulates intestinal Treg production in mouse models of peanut and food allergy, demonstrating anti-inflammatory effects (Atarashi et al., 2013; Barletta et al., 2013; Li et al., 2020; Shi et al., 2020). Furthermore, *Lactobacillus* supplementation is found to reduce airway inflammation in murine model of both asthma (Atarashi et al., 2013; Barletta et al., 2013), and food allergy [45,46), inducing the suppression of type 2 cytokines and enhancing Treg production. This immunomodulatory effect is also seen in human studies; treatment of lactic acid bacteria including *Lactobacillus lactis, Lactobacillus casei, Lactobacillus plantarum* and *Lactobacillus GG* to dendritic cells isolated from allergic patients, significantly induced IL-10 and IL-12 production, indicating enhanced Treg differentiation, and suppressed type 2 cytokines following allergen challenge (Mohamadzadeh et al., 2005). Moreover, when co-cultured with naive CD4+ T cells, Lactobacillus casei treatment significantly increased the production of IFN-y in dendritic cells isolated from asthmatic patients (Ratajczak et al., 2007), a Th1 cytokine which has powerful inhibitory effects on Th2 cell activation, thus regulating T helper cell balance. Taken together, probiotic bacteria are able to regulate T cell differentiation away from Th2 polarization and towards Treg production via activation of TLR signalling, consequently promoting immunotolerance to antigens.

Despite this, it is important to note that the longstanding the Th2-centered paradigm of allergy is somewhat simplistic and reductionist. It is now known that varying endotypes of allergic disease result from complex interactions between many heterogeneous T cell subsets, including Th1, Th17, Th22, Th9, Tfh and Tregs as well as supporting immune cells such as ILC2s and neutrophils. For instance, intrinsic atopic dermatitis, a sub-type accounting for approximately 20% of AD cases, is characterized by over-activation of Th1 cells, stimulating TNF-a and IFN-y production, resulting in epithelial cell apoptosis and the manifestation of eczematous skin lesions (Kabashima-Kubo et al., 2012; Tokura et al., 2018). Moreover, while the majority of asthmatic patients present the type 2 asthma phenotype, a smaller percentage are characterized by neutrophilic asthma involving enhanced activation of Th17 and Th1 cells and neutrophilic inflammation (Pelaia et al., 2015; Boonpiyathad et al., 2019). Thus, although Th2 polarization accounts for a significant proportion of allergic inflammation, one should consider allergic immune response beyond the simple Th1/Th2 dichotomy. Therefore, future research should determine the mechanistic role of LBPs on the regulation of many heterogeneous T cell subsets involved in differing allergy endotypes. Nevertheless, LBPs can have powerful impacts on regulating Th1/Th2 balance, ultimately promoting immune tolerance and contributing to allergy protection.

### SCFA-induced immunomodulation

A further mechanism by which LBPs may influence immune response and reduce risk of allergic disease is via the metabolites they produce, namely short-chain fatty acids. *Bifidobacterium*, *Lactobacillus, Bacteroides* and *Clostridia* in particular, ferment undigestible oligosaccharides and proteins in the colon and cecum to produce SCFAs. Accordingly, probiotic supplementation is repeatedly found to increase fecal SCFA concentration in both adults (Gaisawat et al., 2019; Kusumo et al., 2019) and children (Kim et al., 2015; Berni Canani et al., 2016). SCFAs, including acetate, butyrate and propionate, are known to have a variety of beneficial and anti-inflammatory properties including neuroprotection, supporting glucose homeostasis, cardiovascular protection and immunoregulation. SCFAs have been repeatedly linked to allergy protection, with high fecal butyrate concentration associated with reduced risk of eczema at 1 year of age (Kim et al., 2015), and food allergy and asthma at 6 years (Roduit et al., 2019). In addition, butyrate supplementation is found to significantly reduce airway inflammation in a murine model of asthma (Theiler et al., 2019; Vieira et al., 2019) and food allergy (Vonk et al., 2019). Furthermore, allergic infants demonstrate reduced genetic potential for intestinal butyrate production, having a lack of genes encoding for enzymes that ferment undigestible carbohydrates into butyrate, compared to non-allergic counterparts (Cait et al., 2019). Interestingly, formula milk supplemented with *Lactobacillus rhamnosus* is found to significantly increase fecal abundance of SCFA-producing bacteria, as well as fecal butyrate concentration in infants with CMA (Berni Canani et al., 2016). Of note, in this study, the most allergen-tolerant infants showed the largest increase in fecal SCFA concentrations, supporting the mechanistic role of microbial-derived SCFAs in allergy protection.

SCFAs are thought to influence allergy risk via several pathways, including via activation of G-protein coupled receptors 41 and 43 (GPR41/43), found on several immune cells, IECs and lung epithelial cells (Li et al., 2018). SCFA-induced activation of GPR41/43 has anti-inflammatory effects via p38 MAPK signaling pathways (Kobayashi et al., 2017). For instance, propionate treatment was found to ameliorate airway inflammation in a murine model of asthma via activation of GPR41 pathways (Trompette et al., 2014). Similarly, in a mouse model of CMA, Lactobacillus acidophilus supplementation not only alleviated allergic inflammation, but also increased fecal SCFA concentration and stimulated activation of GPR41 and GRP43 receptors (Wang et al., 2019), emphasizing the pro-tolerogenic effects of probiotic bacteria via SCFA-induced GPR41/43 activation.

In addition, SCFAs may also provide allergy-protective effects via inhibition of histone deacetylase (HDAC). HDACs are enzymes involved in the deacylation of histone proteins, and are key regulators of T cell differentiation (Haery et al., 2015), thus play a key role in allergic immune response. In allergic individuals, allergen exposure up-regulates HDAC expression, stimulating activation of the mTOR-S6K signaling pathway, resulting in elevated IL-4 and IL-6 production, and consequently Th2 and Th17 cell differentiation. In addition, expression of HDACs suppresses FoxP3 transcription in CD4+ T cells, resulting in decreased Treg production (Deng et al., 2020; Lee et al., 2022), contributing to the T cell imbalance seen in allergy. Accordingly, patients with allergic rhinitis (Wang et al., 2015) and asthma (Butler et al., 2012) show increased HDAC1 expression compared to healthy counterparts. Notably, microbial-derived SCFAs, particularly propionate and butyrate, are powerful HDAC inhibitors (Lin et al., 2015). SCFAs are able to cross the intestinal epithelial barrier and inhibit HDAC expression in intestinal epithelial and immune cells, thus up-regulating FoxP3 transcription, suppressing IL-4 and IL-6 production, and ultimately promoting the proliferation of anti-inflammatory Treg cells (Arpaia et al., 2013; Furusawa et al., 2013). In support of this, butyrate supplementation reduces airway inflammation in a murine model of ILC2-driven asthma, inducing IL-13, IL-5 and ILC2 suppression via HDAC inhibition, and independently of GPR41/43 activation (Thio et al., 2018). In addition, the authors reported murine supplementation of butyrate-producing *Clostridia* significantly increased pulmonary propionate and butyrate levels and alleviated asthma. Moreover, another group demonstrated that acetate supplementation promoted the generation of Treg cells and alleviated asthma in a murine model via HDAC inhibition and FoxP3 acetylation (Thorburn et al., 2015). Hence, LBPs may exhibit allergy-protection through SCFA-induced HDAC inhibition, thus regulating T cell balance.

Taken together, LBPs may exhibit allergy protection via the production of microbial-derived metabolites SCFAs, which promote Treg production and immunotolerance through both GPR41/43 signaling pathways and HDAC inhibition. Of note, if the allergy-protective effects of LBPs derive from enhanced production of SCFAs, then future research may also investigate the potential for direct SCFA supplementation as a ‘post biotic’ metabolite for the prevention and treatment of allergic disease. The direct use of SCFA metabolites may bypass certain practical limitations of LBPs, such as cultivation and storage of live organisms.

### Strengthening epithelial barrier integrity

Alongside their immunomodulatory actions, LBPs may also influence allergy susceptibility through physiological mechanisms, such as strengthening epithelial barrier integrity. During allergic sensitization, an otherwise harmless antigen, crosses the mucosal barrier and is presented by antigen-presenting cells, triggering Th2 cell activation and the type 2 inflammatory cascade, ultimately resulting in allergy development (Caminati et al., 2018). Thus, epithelial barriers act as protection preventing certain antigens to cross, enter systematic circulation and interact with immune cells. Accordingly, epithelial barrier dysfunction is commonly associated with allergic disease (Gon and Hashimoto, 2018; Kortekaas Krohn et al., 2020), and increased intestinal barrier permeability is a hallmark feature of food allergy (Parrish et al., 2022). Notably, many probiotic species are able to support the integrity of the intestinal epithelial barrier by stimulating mucus production and stabilizing epithelial tight junctions. For instance, several *Lactobacilli strains* are found to up-regulate MUC3 expression in intestinal epithelial cells, resulting in increased mucus production (Mattar et al., 2002; Das et al., 2016). Similarly, *Streptococcus thermophilus* and *Lactobacillus acidophilus* treatment in HT29 intestinal epithelial cells (IECs) was found to improve epithelial barrier properties by increasing the expression of tight junction proteins actinin and occludin and improving transepithelial resistance (TER), which subsequently protected IECs from *E.coli*-induced damage (Resta-Lenert and Barrett, 2003), highlighting the powerful role of LBPs in strengthening epithelial barrier integrity.

Moreover, alongside their direct effects on epithelial barrier function, LBPs may also act indirectly on the intestinal epithelial barrier via the metabolites their produce, specifically SCFAs. Microbial-derived SCFAs, are known to play a protective role in maintaining epithelial barrier integrity, by up-regulating the expression of MUC2 in IECs, thus stimulating mucus secretion (Burger-van Paassen et al., 2009; Giromini et al., 2022). In addition, SCFAs are found to maintain tight junctions through enhancing the expression of tight junction protein-related genes, GJA3 and occludin (Gao et al., 2021). Therefore, not only may LBPs directly promote intestinal barrier maturation, but also indirectly via their resulting metabolites.

Collectively, these findings indicate that LBPs may confer allergy protection by promoting intestinal barrier maturation and mucosal homeostasis, which may prevent leakage of antigens through the mucosa, reduce allergenic exposure to APCs, and consequently protect against sensitization in the gut. Despite this, further research is needed to confirm the importance of epithelial barrier integrity in allergy development in order to validate this mechanism.

Although the discussed pathways represent the principal mechanisms by which LBPs may influence allergic immune response, this is not an exhaustive list. Other potential mechanisms to consider include IgA-induced regulation of mucosal homeostasis and allergen neutralization (Scheurer et al., 2023), and direct inhibition of mast cell degranulation (Oksaharju et al., 2011; Forsythe et al., 2012; Forsythe, 2016).

## Current findings on the role of LBPs in allergy

### Animal data

A multitude of animal studies demonstrate beneficial immunomodulatory effects of LBPs on allergic inflammation, as summarized in **Table 1**. For instance, *Lactobacillus rhamnosus GG* supplementation was found to alleviate airway hyper-responsiveness, suppress Th2 cytokine release, and decrease immune cell infiltration in the lung, thus dampening Th2 cell response in a murine model of asthma (Wu et al., 2019). In a food allergy model, mono-colonization of Clostridia in germ-free mice not only inhibited sensitization to food allergens, but also increased CD4+Foxp3+ Treg cell numbers, IgA and IL-22 production in the lamina propria, ultimately restoring intestinal epithelial integrity (Stefka et al., 2014), highlighting the pro-tolerogenic properties of Clostridia and the potential mechanistic role on epithelial barrier integrity. Furthermore, four bacterial genera (*Faecalibacterium, Lachnopsichia, Rothia and Vieonella*) found to be significantly decreased in abundance in asthmatic children compared to non-allergic counterparts, were reported to reduce airway inflammation in a murine model of asthma, indicating powerful asthma-protective properties (Arrieta et al., 2015). Similarly, a microbial consortium of seven strains of *Bifidobacteria* and lactic acid bacteria significantly reduced serum IgE, Th2 cell cytokines, and AD-like skin lesions in a mouse model of atopic dermatitis (Shin et al., 2016). Notably, the immunological alleviation of AD was thought to reflect LBP-induced production of CD4+Foxp3+ Treg cells, providing an immunoregulatory effect on Th2 cell polarization. Moreover, Shi et al., 2023 (Shi et al., 2023), demonstrated that *Lactobacillus rhamnosus* administration significantly reduced allergic inflammation in an OVA model of food allergy, including reductions in OVA-specific IgE, histamine and murine mast cell protease 1 (mMCP1), and regulation of Th1/Th2 balance. In addition, fecal SCFA concentration increased following *Lactobacillus* supplementation, supporting the mechanistic role of microbial-derived SCFAs in allergy protection (Shi et al., 2023).

**Table 1:** Summary of mouse studies assessing the therapeutic impact of LBPs in allergic disease.

Reference	Model	Bacterial strain	Treatment duration	Treatment outcome
(Barletta et al., 2013)	Peanut allergy model, peanut extract sensitization	VSL#3 mixture containing *Lactobacillus acidophilus*, *Lactobacillus plantarum*, *Lactobacillus casei*, *Lactobacillus delbrueckii* subspecies *bulgaricus*, *Bifidobacterium breve, Bifidobacterium longum*, and *Bifidobacterium infantis*, and *Streptococcus salivarius* subspecies *thermophilus*	Daily treatment for 20 days in adult mice.	- Ameliorated anaphylaxis symptoms - Supressed Th2 inflammation - Induced TGF-B activation and production of FOXP3 expressing Treg cells
(Shi et al., 2023)	Food allergy model, ovalbumin sensitization	*Lactobacillus rhamnosus* Probio-M9	n/a	- Reduced OVA-specific IgE, histamine and mMCP-1 - Dampened Th2 inflammation - Increased faecal abundance of *Firmicutes Bacteroidota* - Increased faecal SCFA levels
(Shin et al., 2016)	Atopic dermatitis model, 1-chloro-2,4-dinitrobenzene sensitization	*Multi-strain consortium containing: Lactobacillus acidophilus* CBT LA1, *Lactobacillus rhamnosus* CBT LR5, *Lactobacillus plantarum* CBT LP3, *Bifidobacterium bifidum* CBT BF3, *Bifidobacterium breve* CBT BR3, *Lactococcus lactis* CBT SL6, and *Streptococcus thermophilus* CBT ST3	Daily treatment for 8 weeks in adult mice	- Alleviated atopic dermatitis skin lesions - Reduced serum IgE levels - Reduced type 2 cytokines - Elevated proportion of CD4^+^Foxp3^+^ Tregs
(Wu et al., 2019)	Asthma model, ovalbumin sensitization	Lactobacillus *rhammosus GG* ATCC53103	Mice were either treated with *L. rhamnosus* a) prior to allergic sensitization daily from day 1-14, and 45-60, or b) post allergic sensitization daily from days 14-28 and 60-75	- Alleviated airway hyper-responsiveness - Supressed Th2 cytokines in BALF, as well as IL-17, TNF-α and HMGB1 - Reduced levels of infiltrating immune cells in BALF including eosinophils, lymphocytes, neutrophils and monocytes.
(Stefka et al., 2014)	Food allergy model, peanut extract sensitization, germ-free mice	Clostridia isolated from murine faecal samples	Administered twice, once before weaning and once two weeks after	- Inhibited sensitization to allergen - Increased CD4+Foxp3+ Treg cell numbers - Elevated IgA and IL-22 production in the lamina propria
(Arrieta et al., 2015)	Asthma model, ovalbumin sensitization	Multi-strain consortium consisting of *Feacalibacterium prausnitzii* ATCC 27766, *Veillonella parvula* ATCC 10790*, Rothia mucilaginosa* ATCC 49040 and *Lachnospira multipara* DSM-3073	Adult mice treated with microbial mixture on days 1, 7 and 14, then paired for breeding. Asthma model ran in offspring.	- Alleviated airway inflammation as measured by histological scoring of lung tissue - Reduced proinflammatory cytokines in lung tissue including IL-6, IL-17A, INF-y and TNF-a - Supressed OVA-specific IgE levels in serum - Decreased immune cell infiltration in BALF, including neutrophils and lymphocytes - Elevated faecal SCFA concentration
(Neau et al., 2016)	Cow’s milk allergy model, β‐lactoglobulin sensitization	*Bifidobacterium animalis* subsp. *lactis* P17, *Bifidobacterium animalis* subsp. *lactis LA*306, *Bifidobacterium bifidum* P122, *Lactobacillus rhamnosus* LA305, *Bifidobacterium longum* subsp. *infantis* LA308, *Lactobacillus salivarius* LA307	Daily supplementation of 1 of the selected bacterial strains alone for 41 days in 4-week-old mice.	- Out of the 6 strains assessed, *L. rhamnosus* LA305, *L. salivarius* LA307, and *B. longum* subsp. *infantis* LA308 significantly reduced serum IgE levels, protected against allergic sensitization and reduced mast cell degranulation - *Lactobacillus rhamnosus* LA305 induced Th1 and Treg responses - *Bifidobacterium longum* subsp. *infantis* LA308 induced Th1 responses - The remaining 3 strains had no impact on allergic inflammation
(Wang et al., 2019)	Cow’s milk allergy model, β‐lactoglobulin sensitization	*Lactobacillus acidophilus* KLDS 1.0738	Administered 3 times per week for 4 weeks	- Supressed hypersensitivity allergic response - Reduced IL‐4, IL‐6, IL‐17, and TNF‐β production, and increased anti-inflammatory cytokines, IFN‐γ and TGF‐β - Increased faecal SCFA concentration - Activated SCFA receptors, GPR41 and GPR43, in spleen and colon
(Feehley et al., 2019)	Cow’s milk allergy model, β‐lactoglobulin sensitization	Fecal microbiota transplant (FMT) from either a) infants with cow’s milk allergy or b) healthy controls, into germ-free mice	One-time FMT	- Mice colonized with FMT from healthy donors were protected from anaphylactic responses, whereas colonization with CMA-infant derived FMT induced drops in core body temperature and clinical manifestations of anaphylaxis in mice. - Significant differences were found between both microbiota composition of cow’s milk allergic vs healthy control humans, and in mice. -Unique transcriptome signatures in the ileal epithelium were found between healthy and CMA colonized mice - *Anaerostipes caccae* specifically was correlated with protection against an allergic response to cow’s milk allergen
(Mauras et al., 2019)	Cow’s milk allergy model, β‐lactoglobulin sensitization	FMT from either a) infants with cow’s milk allergy or b) healthy controls, into germ-free mice	One-time FMT in 3-week-old mice	- Mice treated with FMTs from CMA-infants showed increased clinical scoring of CMA including diarrhoea, skin puffiness/scratching and anal inflammation, compared to mice colonized with healthy-donor FMT. - Mice colonized with FMT from CMA-infants showed elevated serum IgE and colonic gata3 mRNA (marker of Th2 cell activation), compared to mice treated with FMT from healthy infants. - Treatment with healthy-donor FMT significantly increased *Bifidobacteria/Lachnospiraceae* ratio in mice. - Specifically, an enrichment of *Bifidobacterium* and *Anaerostipes* (butyrate-producing) was found in mice colonized with healthy-donor FMTs and protected from allergic response.

n/a, unknown or unavailable.

Furthermore, a handful of animal studies have assessed the efficacy of fecal microbiota transplantation (FMT) for the treatment of allergic disease, demonstrating promising results.

Feehley et al., 2019 (Feehley et al., 2019) reported that treatment of germ-free mice with FMTs derived from healthy infants protected against anaphylactic responses to cow’s milk allergen, whereas FMT from infants with CMA resulted in reductions in core body temperature, indicating anaphylactic allergic response. Accordingly, significant differences in fecal microbiota composition were observed between both infants with CMA compared to healthy controls, and the respectively colonized mice. Similarly, Mauraus et al., 2019 (Mauras et al., 2019) demonstrated that FMT derived from CMA-infants significantly increased clinical manifestations of food allergy in sensitized germ-free mice, including diarrhea, skin puffiness/scratching and anal inflammation, as well as serum IgE levels and colonic gata3 mRNA (a marker of Th2 cell activation), compared to mice treated with FMT from healthy infants. Notably, the observed allergy-protection following healthy-donor FMT was associated with increased *Bifidobacteria/Lachnospiraceae* ratio including enhanced abundance of *Anaerostipes* (Mauras et al., 2019), indicating an allergy-protective role of this butyrate-producing genus and hinting to underlying SCFA-dependent mechanisms. Collectively, these findings suggest encouraging potential for harnessing FMT as a therapeutic tool for allergic disease. However, it is important to note that data of FMTs in allergy is limited and preliminary, with only a handful of small-scale animal studies conducted to date. Therefore, substantially more research is needed, with several challenges yet to be overcome before the therapeutic efficacy of FMT in allergy can be assessed in human trails.

Despite some promising results, it should be noted that contrasting animal findings demonstrating no beneficial effects of LBPs on allergy are also reported, although more sparse. For instance, Neau et al., 2016 found that *Bifidobacterium animalis* subsp. *lactis* had no impact on Th2 inflammation in a mouse model of CMA, yet *Bifidobacterium longum* subsp. *infantis* did (Neau et al., 2016), indicating strain-specific effects. Nevertheless, taken together, animal data suggests a powerful immunomodulatory role of microbial intervention in the prevention and management of atopic disease, indicating promising potential of LBPs. However, despite this, data from human trials is much more inconsistent; The more positive findings from animal studies likely reflects the greater number of cofounding variables, population heterogeneity, and complexity found in human trials, which are limited in animal research.

### Human data

Given our ever-evolving understanding of the tightly intertwined relationship between the gut microbiota and immune maturation, an increasing number of randomized controlled trails (RCTs) have assessed the role of various LBPs in the prevention and treatment of allergic disease, as shown in **Table 2**. While findings are inconsistent, several studies indicate promising potential for the therapeutic value of LBPs in childhood allergy. In terms of allergy prevention, maternal supplementation of either a) *Lactobacillus rhamnosus* LPR and *Bifidobacterium longum* BL999 or b) *Lactobacillus paracasei* ST11 and B longum BL999 during the last two months of pregnancy and first two months of breastfeeding was found to significantly reduce the incidence of atopic dermatitis in infants at 2 years of age, indicating powerful allergy-preventive effects (Rautava et al., 2012). However notably, these bacteria had no effect on risk of atopic sensitization to a panel common food and plant allergens, suggesting the benefit is disease-specific. Similarly, in a RCT, maternal pre- and post-natal supplementation of *Lactobacillus rhamnosos GG, L. acidophilus* and *Bifidobacterium animalis subsp. lactis* lead to significant reductions in the incidence of atopic dermatitis in offspring (followed to 6 years of age), compared to control (Simpson et al., 2015). However, no effect was found on childhood incidence of atopic sensitization, asthma and allergic rhinoconjunctivitis, again indicating the protective efficacy is disease-specific. Accordingly, the World Allergy Organization (WAO), recommends probiotic supplements for pregnant and breastfeeding women, and infants, at high-risk of allergy, for the prevention of atopic dermatitis (Fiocchi et al., 2015). This clinical recommendation however, does not currently extend to the prevention of other atopic diseases, due to insufficient clinical data.

**Table 2:** Summary of human randomized controlled trials assessing the therapeutic impact of LBPs in allergic disease.

*References*	Allergy assessed	Population	Bacterial strain	Treatment duration	Treatment outcome
(Rautava et al., 2012)	Eczema Atopic sensitization	205 mother-infant dyads	Either: a) *Lactobacillus rhamnosus* LPR and *Bifidobacterium longum* BL999 Or b) *L paracasei* ST11 and *B longum* BL999	Maternal daily supplementation from last 2 months of gestation through till 2 months postpartum	- Supplementation of both probiotic treatments significantly reduced the risk of eczema development during the first year of life - No effect on risk of atopic sensitization in infants
(Simpson et al., 2015)	Atopic dermatitis Asthma Allergic rhinoconjunctivitis, Atopic sensitization	163 mother-infant dyads	Milk containing *Lactobacillus rhamnosos GG, L. acidophilus La-5* and *Bifidobacterium animalis* subsp. *lactis*	Maternal daily supplementation from 36 weeks of gestation till 3 months postpartum	- Reduced the incidence of atopic dermatitis in children at 6 years of age, compared to control - No impact on the risk of asthma, allergic rhinoconjunctivitis, or atopic dermatitis at 6 years old
(Canani et al., 2012)	Cow’s milk allergy (CMA)	55 infants aged 1-12 months with CMA	*Lactobacillus rhamnosus GG-* supplemented formula milk	Daily consumption of *Lactobacillus rhamnosus GG-*supplemented formula milk, containing 1.46 × 10^7^ CFU/100 mL, for 12 months	- Significantly improved tolerance to cow’s milk challenge in infants with CMA, at 6 and 12 months follow up - Enhanced cow’s milk tolerance was associated in shifts in microbiota composition to increased abundance of *Blautia, Roseburia, Coprococcus* and *Oscillospir*a
(Strisciuglio et al., 2023)	Cow’s milk allergy	8 infants aged 6-12 months with CMA	Consortium of *Bifidobacterium Longum* BB536*, Bifidobacterium infantis *M-63 *Bifidobacterium breve *M-16	Daily treatment for 45 days	- Bifidobacterium treatment significantly decreased the proportion of naïve T cells, activated CD4+ T, Th2 activation and basophil degranulation in blood, compared to control, indicating immunotolerance. - Probiotic-induced immunomodulation persisted after 45-day wash-out period
(Chen et al., 2010)	Asthma Allergic rhinitis (AR)	105 children aged 6-12 years old	*Lactobacillus gasseri* A5	Daily supplementation for 8 weeks	- Significantly decreased clinical symptom scoring of asthma and atopic rhinitis - Treatment improved pulmonary function and peak expiratory flow rates (PEFR), compared to control - Significant reductions in TNF-α, IFN-γ, IL-12, and IL-13 production from HDM-stimulated PBMCs isolated from allergic children
(Abrahamsson et al., 2013)	Asthma Allergic rhinoconjunctivitis Eczema Atopic sensitization	184 mother-infant dyads, with a family history of allergy	*Lactobacillus reuteri* 57730	Maternal daily supplementation from 36 weeks gestation, followed by infant supplementation from birth-12 months of age	- *L. reuteri* supplementation had no impact on risk of asthma, allergic rhinoconjunctivitis, eczema or atopic sensitization during 7 year follow up - Decreased Th2 and Th1 cytokines at 6 months of age, however this effect did not persist any later
(Ou et al., 2012)	Atopic sensitization Asthma Atopic dermatitis Allergic rhinoconjunctivitis	191 mother-infant dyads	*Lactobacillus rhamnosus GG* 53103	Maternal treatment of 1x10^10^ CFU daily from 24 weeks of gestation till 6 months postpartum	- No impact on risk of any allergic disease assessed in children infants followed till 36 months of age - Significantly reduced maternal allergic scoring, particularly in mothers with IgE >100 kU/L - Improvement in maternal allergy symptoms was accompanied by an increase in plasma IL-12
(Prescott et al., 2008)	Atopic dermatitis IgE-mediated food allergy Asthma Atopic sensitization	153 infants followed from birth- 2.5 years of age, born from mothers with allergic disease	*Lactobacillus acidophilus* L10/LAVRI-A1	3x10^9^ CFU daily from birth- 6 months of age	- No impact on risk of atopic dermatitis, IgE-mediated food allergy, asthma, or atopic sensitization at 2.5 years of age
(Marlow et al., 2015)	Atopic dermatitis	331 mother-infant dyads	Either a) *Lactobacillus rhamnosus HN001* or b) *Bifidobacterium animalis subsp. lactis HN019*	Maternal supplementation of either a) or b) from 35 weeks gestation till 6 months postpartum in breastfeeding mothers. All infants received daily supplementation corresponding to maternal treatment, from birth- 2 years of age.	- Toll-like receptor (TLR) genotypes of the infant influenced both pre-disposition to eczema and efficacy of probiotic treatment in reducing eczema development - 26 TLR single nucleotide polymorphisms (SNPs) interacted with *Lactobacillus rhamnosus* reduced risk of eczema - 2 TLR SNPs interacted with *Bifidobacterium animalis subsp. lactis* resulting in reduced risk of eczema, eczema severity or atopy
(Wang and Wang, 2015)	Atopic dermatitis	210 children aged 1-18 years old with atopic dermatitis	Either: a) *Lactobacillus paracasei* GMNL-133, b) *Lactobacillus fermentum* GM090, or c) *Lactobacillus paracasei* GMNL-133 and *Lactobacillus fermentum* GM090 combined	Daily supplementation of the following for 3 months: either *a) Lactobacillus paracasei* GMNL-133, b) *Lactobacillus fermentum* GM090 *or c) Lactobacillus paracasei* GMNL-133 and Lactobacillus *fermentum* GM090 combined	- Supplementation of *L.fermentum* and *L.paracasei* combined significantly reduced atopic dermatitis severity, as measured by SCORAD scoring. This benefit remained at 4 months post probiotic cessation - All treatment groups showed reductions in serum IgE, IL-4, TNF-α, urine eosinophilic protein X, and 8-OHdG levels, and increases in I FN-γ and TGF-β
(Yan et al., 2019)	Atopic dermatitis	Infants aged 4-30 months with atopic dermatitis	Heat-treated *Lactobacillus paracasei GM-080*	Daily supplementation for 16 weeks	- *L.paracasei* supplementation had no impact on atopic dermatitis severity, including SCORAD, itching, and IDQOL, compared to control
(Gore et al., 2012)	Atopic dermatitis	137 infants aged 3-6 months with eczema	Either:*a) Lactobacillus paracasei* CNCM I-2116 or b) *Bifidobacterium lactis* CNCM I- 3446	Daily supplementation of either *Lactobacillus paracasei* or *Bifidobacterium lactis* for 3 months	- No significant differences were found in eczema severity between probiotic and control treated infants
(Niers et al., 2009)	Eczema	123 mother-infant dyads with a family history of allergy	Consortium of *Bifidobacterium bifidum* W23, *Bifidobacterium lactis* W52, and *Lactococcus lactis* W58	Maternal daily supplementation for last 6 weeks of pregnancy, and infant supplementation daily from birth- 1 year of age.	- Significantly reduced the risk of eczema development within the first 3 months of life - After 3 months of age, eczema incidence was similar in probiotic and control groups - Probiotic-induced eczema prevention during first 3 months of life was sustained at 2 years of age - Probiotic treatment significantly decreased IL-5 levels in blood, compared to control
(Gorissen et al., 2014)	Asthma Allergic rhinitis	83 children from the above study (Abrahamsson et al., 2013) followed up at 6 years of age	Consortium of *Bifidobacterium bifidum* W23, *Bifidobacterium lactis* W52, and *Lactococcus lactis* W58	Maternal daily supplementation for last 6 weeks of pregnancy, and infant supplementation daily from birth- 1 year of age.	- Follow up at 6 years of age found probiotic supplementation had no impact on the development of asthma or allergic rhinitis
(Huang et al., 2018)	Asthma	160 children aged 6-18 years old with asthma	Either: a) *Lactobacillus paracasei* GMNL-133, b) *Lactobacillus fermentum* GM090, or c) *Lactobacillus paracasei* GMNL-133 and *Lactobacillus fermentum* GM090 combined	Daily supplementation of the following for 3 months: either *a) Lactobacillus paracasei* GMNL-133, b) *Lactobacillus fermentum* GM090 *or c) Lactobacillus paracasei* GMNL-133 and Lactobacillus *fermentum* GM090 combined	- All probiotic-treated groups showed significant reductions in asthma severity - Supplementation of *L.paracasei* and *L.fermentum* combined significantly improved PEFR scores and decreased serum IgE levels
(Rose et al., 2010)	Asthma Atopic sensitization	131 infants aged 6-24 months old with a family history of allergy and two episodes of physician-diagnosed wheezing	*Lactobacillus rhamnosus* GG	Twice daily supplementation of 10^10^ CFU of *L.rhamnosus* GG for 6 months	- No difference in the incidence of atopic dermatitis or asthma-related events in children between probiotic-treated and control group - Probiotic treatment showed mild immunomodulatory effects on atopic sensitization: IgE specific for aeroallergens was reduced in probiotic-treated children compared to control
(Giovannini et al., 2007)	Asthma Allergic rhinitis	187 children aged 2-5 years old with asthma and/or allergic rhinitis	*Lactobacillus casei-* supplemented milk	Daily supplementation of 10^8^ CFU for 12 months	- No effect on asthma outcomes in children, including number or asthma episodes, severity questionnaires and serum IgE - In allergic rhinitis, probiotic treatment significantly decreased the number of AR episodes reported, and duration of diarrhoea episodes - No benefit on serum IgE levels in children with AR

In terms of treatment of existing allergy, *Bifidobacterium bifidum* supplementation significantly reduced food allergy symptoms and serum IgE levels, and elevated serum IgG2, anti-inflammatory responses and restore gut microbiota composition in a RCT of infants aged 1–12 months with cow’s milk allergy (CMA) (Jing et al., 2020). Moreover, *Lactobacillus rhamnosus* GG-supplemented formula milk was found to improve tolerance acquisition in infants with CMA (Canani et al., 2012). Notably, enhanced cow’s milk tolerance was associated with shifts in intestinal microbiota composition, including increased abundance of *Blautia, Roseburia, Coprococcus* and *Oscillospira* following *Lactobacillus rhamnosus GG* supplementation, all of which are known SCFA producers, as well as significant increases in fecal butyrate production (Berni Canani et al., 2016). Thus, these findings suggest that LBP supplementation may improve antigen tolerance through shifting microbiome composition towards increased abundance of SCFA-producing bacteria, supporting the mechanistic role of SCFAs in allergy protection. Similarly, supplementation with *Bifidobacterium Longum* BB536, *Bifidobacterium Infantis* M-63 and *Bifidobacterium breve* promoted oral tolerance to cow’s milk, as well as reduced circulating CD4+ cells, Th2 cell activation and basophil degranulation in infants aged 6–12 months with CMA (Strisciuglio et al., 2023), highlighting the powerful immunomodulatory role of *Bifidobacteria* in suppressing Th2 cell polarization. Interestingly in this study, immunotolerance to cow’s milk persisted beyond both the LBP treatment period and 45-day wash-out period, suggesting that LBP-induced immunomodulation during the early-life critical window of immune development has lasting effects on immunotolerance to antigens beyond treatment period. In terms of asthma, *Lactobacillus gasseri* treatment was found to significantly improve peak excitatory flow rates (PEFR) and asthma severity scores, and decrease TNF-a, IFN-g, IL-12, and IL-13 release from HDM-stimulated PBMCs in asthmatic children, compared to control (Chen et al., 2010). Accordingly, LBP’s may demonstrate powerful pro-tolerogenic effects in existing allergy, providing significant therapeutic value for allergy management.

However, despite some promising findings on the role of LBPs in allergy prevention and management, data is inconsistent, and many contrasting studies demonstrate no protective effect on allergy outcomes. For instance, maternal and infant supplementation of *Lactobacillus reuteri* throughout the last month of gestation and first year of life, had no impact on the incidence of atopic sensitization, IgE-associated eczema or asthma in children at high risk of allergy, followed till 6 years of age (Abrahamsson et al., 2013). Moreover, maternal supplementation of *Lactobacillus GG* during pregnancy till 6 months postpartum had no effect on the development of sensitization or allergic disease in offspring at 3 years of age (Ou et al., 2012). However, interestingly in this study, although maternal *Lactobacillus GG* supplementation did not reduce risk of allergy in offspring, a reduction in the severity of maternal allergic disease was found, indicating LBP’s potential for allergy-protective immunomodulation in adults, beyond the early-life period. Furthermore, *Lactobacillus acidophilus* treatment for the first 6 months of life had no impact on susceptibility to atopic sensitization or allergic disease at 2.5 years of age in infants at high risk of allergy (Prescott et al., 2008). Inconsistency in findings likely reflects a multitude of factors including heterogeneity in bacterial strains assessed, timing, dosage and duration of supplementation, follow-up periods evaluated and population variables such as genetics, geographical location and individual allergy risk. Such complex interplay between many influencing factors makes it difficult to compare across human trials and draw conclusions on the efficacy of LBPs in the prevention of allergic disease.

### Critical evaluation of current research

Given the plethora of factors which influence infant microbiota colonization, these likely also impact the therapeutic efficacy of LBPs in childhood allergy, reflecting such inconsistent findings from human trials. Therefore, establishing the overall allergy-protective value of LBPs for the general population is highly complex and nuanced. Several issues and methodological limitations in the current research likely contribute to contrasting results and should be considered in future studies.

Firstly, the allergy-protective efficacy of LBPs may vary depending on geographical location and population genetics; For instance, a meta-analysis of RCTs reported that Asian children aged 1–18 years old showed reduced AD severity (as measured by SCORAD) following *Lactobacillus* supplementation, whereas this LBP had no impact on allergy outcomes in European populations (Huang et al., 2017). The fact that the same treatment can have differential effects on allergy outcomes between varying populations may be explained by a plethora of factors that influence microbiota and immune development, such variation in diet, microbial exposures, genetics, and lifestyle. In a similar vein, Marlow et al., 2015 (Marlow et al., 2015) demonstrated that the impact of *Lactobacillus rhamnosus* and *Bifidobacterium animalis subsp. lactis* on eczema and atopy outcomes was dependent on variations in TLR genetic polymorphisms of the infants. Here, specific TLR genotypes influenced both the infants pre-disposition to atopy and the efficacy of the LBP supplementation, suggesting the allergy-protective effects of LBPs may vary depending on individual’s genetics. Considering this, future RCTs should assess the therapeutic value of LBPs across varying geographical locations and genotypes to determine their applicability amongst a range of heterogenous populations.

Moreover, heterogeneity between bacterial strains assessed, makes it difficult to compare across studies and draw conclusions. The efficacy of LBPs is highly species and strain-specific, thus adding to inconsistency in findings. For instance, *Lactobacillus paracasei* GMNL-133 supplementation was found to significantly reduce severity of atopic dermatitis, as measured by SCORAD, in children aged 1–18 years, providing a therapeutic benefit which continued beyond the treatment period (Wang and Wang, 2015). However in contrast, supplementation of differing strains of the same species, *Lactobacillus paracasei* GM-080 (Yan et al., 2019) and *Lactobacillus paracasei* CNCM I-2116 (Gore et al., 2012), had no impact on atopic dermatitis severity, thus demonstrating strain-specific variation in the allergy protective efficacy of this species. Although methodological and population differences between studies may contribute to these contrasting findings, strain-specific variation should be considered when evaluating the therapeutic value of LBPs in allergy. Therefore, future research should compare the therapeutic benefits of varying standardized bacterial strains across a range of allergic diseases, to determine optimal strains for allergy protection.

Furthermore, it is important to consider the timing of LBP supplementation, as therapeutic benefits may vary depending on when the supplementation is administered during the perinatal period. For instance, a 2016 meta-analysis reported that the preventive efficacy of LBPs on allergy development was highest when supplemented to both the mother-infant dyad pre- and post-natally, whereas infant supplementation alone had no overall impact on allergy outcomes (Wang et al., 2016), suggesting that microbial exposure during the prenatal period is important for fetal immune development and subsequent immunotolerance. However in contrast, another meta-analysis examining the role of LBPs on asthma risk revealed that only neonatal probiotic supplementation reduced asthma risk, whereas maternal combined pre- and post- natal supplementation did not (Du et al., 2019). As immune development begins in-utero, and maternal microbial exposure during pregnancy is known to influence fetal immune programming via transplacental transmission (Mishra et al., 2021; Husso et al., 2023), it is likely that initiation of probiotic intervention during pregnancy (and continued throughout early infancy), may confer the most immunotolerance to offspring. Nevertheless, greater research is necessary to compare timing and duration of LBP supplementation and determine the optimal supplementation regime to yield the greatest allergy protection in infants.

Moreover, the therapeutic value of LBPs in allergy prevention may depend on the child’s risk status and pre-disposition to atopy development. Several trials report that LBP supplementation is most effective at reducing risk of allergy development in populations that are already pre-disposed to increased risk of atopic disease e.g., aforementioned TLR polymorphisms (Marlow et al., 2015) and family history of atopy. For instance, a meta-analysis demonstrated that pre- and post-natal probiotic supplementation had greater efficacy in reducing allergy and food hypersensitivity susceptibility in families at high risk of allergy, compared to those with no predisposition (Zhang et al., 2016). These findings are applicable for informing future clinical recommendations of LBP interventions for mother/infants with a known risk of allergy. In addition, future research should examine the impact of LBPs on allergy outcomes of children with a low-risk status, to determine their value to the general population.

In addition, when evaluating LBP efficacy, it is important to recognize the method of LBP administration and possible synergistic effects with other nutrients. For instance, breast milk contains human milk oligosaccharides (HMOs) which have powerful prebiotic properties and are known to support the growth of many intestinal commensal bacteria (Musilova et al., 2014; Salli et al., 2020; Walsh et al., 2020). To this regard, breast milk can be considered a natural ‘synbiotic’ containing both commensal bacteria and prebiotic HMOs to support microbial function. Therefore, the allergy-protective efficacy of LBPs may be influenced by whether infants are formula- or breast- fed. Moreover, aside from breast milk, the type of formula in which infants receive may have a cofounding influence on the colonization stability and efficacy of LBPs; As lactose is known to have a bifidogenic effect (Yılmaz-Ersan et al., 2016; Gallier et al., 2020), the varying concentration of lactose between formula milks, particularly amino-acid based/hydrolyzed vs. regular formula, may influence the stability and function of LBPs in the colon. Accordingly, a meta-analysis found that beneficial effects of LBPs on allergy outcomes were reported more by studies in which infants received amino acid-based or hydrolyzed formula, compared to those receiving standard formula or probiotics alone (Sestito et al., 2020), indicating a possible synergistic effect of amino-acid based/hydrolyzed formulas with LBPs. Consequently, method of feeding, including breastfeeding or formula type, should be taken into consideration when drawing conclusions on the protective role of LBPs in allergic disease.

Furthermore, the lasting protective effects of LBPs on long-term allergy outcomes are unclear. While several studies suggest that LBP supplementation protects against atopic disease throughout childhood, others suggest this protection is short-lived. For instance, Niers et al., 2009 (Niers et al., 2009) demonstrated that pre-and post-natal supplementation of a microbial consortium, consisting of *Bifidobacterium bifidum, Bifidobacterium lactis and Lactococcus lactis*, till 1 year of age significantly reduced incidence of eczema, including reduced IL-5 production, at 2 years old. However, this protective effect was no longer present by 6 years of age (Gorissen et al., 2014), demonstrating only temporary benefits within infancy. Notably, as the first 1000 days of life are considered as the early-life critical window in which microbiota colonization shapes immune system programming (Gensollen et al., 2016; Iyer and Blumberg, 2018; Romano-Keeler and Sun, 2022), microbiota and immune development are still ongoing at 1 year of age (the time of probiotic cessation in this study), thus it is possible that longer-term probiotic intervention throughout the entirety of the critical window is necessary to provide long-lasting allergy protection. In contrast however, other studies have demonstrated long-lasting allergy protection following early-life probiotics; Pre-natal and infant supplementation of *Lactobacillus rhamnosus* from pregnancy till 2 years of age (notably a much longer treatment duration than Neirs et al.,2009 (Niers et al., 2009), was found to significantly reduce both atopic dermatitis and hay fever susceptibility at 11 years of age, indicating long-term protective effects of this LBP (Wickens et al., 2018). Along with this inconsistency, many probiotic trials lack long-term follow-ups, making it difficult to draw conclusions on the long-term protective efficacy of probiotic supplementation beyond infancy. This likely reflects logistical issues in maintaining participant follow up and high dropout rates. Nevertheless, future research should emphasize long-term follow up in order to determine the lasting therapeutic role of early-life LBP supplementation on allergy outcomes throughout the lifespan.

Lastly, various methodological limitations limit current findings and should be addressed in future research; Studies tend to recruit mothers and infants at high risk of allergy, which although has a valid rationale, creates selection bias and makes it difficult to determine the protective efficacy of LBPs against allergic disease in low-moderate risk individuals. In addition, many trials are limited by short follow-up periods, meaning the long-term impact of LBPs on allergy outcomes beyond infancy and throughout life are unclear. Moreover, several studies fail to account for cofounding variables including infant diet, breastfeeding vs. formula feeding, lifestyle factors and external microbial exposures (e.g., cesarean-delivery & antibiotic use), which can impact infant microbiota composition and thus LBP colonization and function. Such limitations should be addressed in robust and well-designed long-term RCTs.

### Future research directions

Despite some promising data indicating an allergy-preventive role of LBPs, findings are limited and inconsistent, thus the WAO conclude that aside from eczema, there is currently not enough supporting evidence to enable clinical recommendations on the use of LBPs for the prevention of childhood allergic disease (Fiocchi et al., 2015). Therefore, greater research in the form of robust RCTs in a variety of heterogeneous populations, is necessary to greater determine the therapeutic applicability of LBPs in a variety of allergic diseases, in order for clinical recommendations to be made.

Regarding future research directions, the optimal bacterial strains, dosage, duration and timing of intervention to best prevent allergic disease are currently unknown, thus further research is necessary to specifically determine the aforementioned criteria. Well-controlled studies should directly compare multiple standardized bacterial strains, dosages and supplementation durations to determine the optimal intervention regime for both allergy prevention and management.

In addition, future RCTs should evaluate standardized atopy assessments, include mother-infant dyads of both high and low allergy risk, involve long-term follow-ups, account for potential synergistic co-founding variables, and assess populations across varying geographical locations and genotypes.

In terms of determining the optimal bacterial strains for allergy protection, the majority of strains assessed thus far are known probiotic species (e.g., lactic acid bacteria), yet further investigation into the compositional differences of the gut microbiota of allergic compared to healthy infants, may provide greater insight into specific ‘allergy protective’ microbes. As it is well established that intestinal microbiota composition during the early-life critical window varies significantly between healthy vs. allergic infants (Kourosh et al., 2018; Boutin et al., 2020; Lee-Sarwar et al., 2023), examination of the specific microbial communities that differ will enable greater understanding of the bacterial strains involved in allergy susceptibility and hint to their use as a LBP. As such, selection of a consortium of bacterial species present in healthy infants, but not allergic, may have greater relevance and efficacy as a therapeutic tool for allergic disease, compared to traditional generalized probiotic strains.

Furthermore, future research should continue to determine the potential therapeutic role of FMT in allergic disease. Currently, FMT is FDA-approved only for the treatment of recurrent *Clostridium difficile* (*C.diff*) infection (US Food and Drug Administration, Fecal FA, 2023), showing well-tolerated and successful results (Hui et al., 2019; Hvas et al., 2019). Given both the efficacy of FMT in *C.diff* infection and preliminary findings from animal models, FMT holds promising potential for an array of other microbiota-associated diseases, including allergy. Accordingly, further investigation to validate the efficacy and safety of FMT in allergy models, as well as enable a greater understanding of what characterizes an ‘allergy-protective’ microbiota composition and the underlying immunological mechanisms, is needed to prior to conduction of clinical trials. In addition, future research should address challenges of donor selection and standardization procedures, along with determining the long-term implications of FMT. With growing acceptance and understanding, FMT offers exciting potential as an alternative therapeutic tool not only for allergy, but also other difficult-to-treat microbiota-mediated and immunological diseases such as autoimmunity, as a means to restore the ‘normal’ microbiota and regulate immune response, thus warrants further exploration.

Moreover, given the established allergy-protective capability of microbial-derived SCFAs (Roduit et al., 2019; Theiler et al., 2019; Vieira et al., 2019; Vonk et al., 2019), combining LBPs with SCFAs, alongside microbial-supporting prebiotics such as HMOs, may aid the immunomodulatory function of LBPs and thus increase their efficacy in allergy prevention. Accordingly, further research should investigate the potential of synbiotic mixtures combining well-researched probiotic strains with prebiotics such as HMOs, GOS or FOS, to aid colonization stability and function. Moreover, future research should assess whether microbial-derived metabolites, SCFAs, which represent a key mechanism by which LBPs modulate immune response, may be used in combination with LBPs as a holistic ‘post-biotic’ to support microbial function and optimize allergy protection.

If SCFAs represent an effective functional metabolite by which LBPs exert anti-allergy effects, then directly harnessing them as a therapeutic tool may also be an exciting avenue for childhood allergy prevention.

Likewise, in addition to SCFAs, future research should also investigate the potential allergy-protective effects of other microbial-derived metabolites. While SCFAs are a predominant and well-established group of anti-inflammatory metabolites, preliminary findings suggest that other metabolites produced by colonic bacteria, such as poly-γ-glutamic acid (γPGA) and tryptophan metabolites, can yield immunoregulatory effects that may offer value as a therapeutic for allergy (Kim et al., 2009; Lee K. et al., 2011; Losol et al., 2024). For instance, γPGA, a metabolite produced predominantly by *Bacillus subtilis* and *Staphylococcus epidermidis* (Jose Anju et al., 2018) is found to have an anti-inflammatory role in atopic dermatitis (AD) through its anti-microbial effects against *Staphylococcus aureus* (Inbaraj et al., 2011; Park et al., 2018), a key species implicated in the development of AD (Kim et al., 2019). Supportingly, murine supplementation of γPGA induced Treg production, regulated Th1/Th2 cell balance, enhanced natural killer cell activity via TLR4 signalling, and suppressed secretion of type 2 cytokines from epithelial cells via GPR-activation, resulting in the prevention of AD-like symptoms in mice (Lee K. et al., 2011; Lee SW. et al., 2013). Similarly, tryptophan metabolites such as indole derivatives and kynurenine produced from microbial metabolism of L-tryptophan, particularly by *Escherichia coli, Clostridium*, and *Lactobacilli* (Williams et al., 2014; Li et al., 2021), are also found to have immunomodulatory effects, including inhibition of mast cell degranulation, as well as regulation of T cell differentiation and dendritic cell immunogenicity (Mezrich et al., 2010; Zelante et al., 2013; Kawasaki et al., 2014; Losol et al., 2024), all of which may promote immunotolerance to antigens. Therefore, emerging data suggests that alongside traditional SCFAs, other microbial-derived metabolites may have notable immunoregulatory effects and hold therapeutic value in allergic disease. However, these findings are preliminary, and significantly more research is needed to determine the long-term efficacy of such metabolites on allergy outcomes, their impact on microbiota composition, and exact immunological mechanisms before translation to human trials. Accordingly, the potential synergistic effects of combining both LBPs and microbial metabolites as a holistic ‘pre- and post-biotic’ to optimize allergy-protection warrants further exploration and holds exciting promise. Moreover, a comparison of the immunomodulatory functions of various microbial metabolites, such as SCFAs, γPGA, indole derivatives, kynurenine, and others, to determine the most effective metabolite for allergy protection, would yield significant value.

In a similar regard, the synergistic effects provided by a consortium of bacterial species combined may increase efficacy against allergy. A meta-analysis of human trials reported that the preventive effects of probiotic supplementation on atopic dermatitis development were most pronounced when a combination of multiple species were administered together, rather than a single species alone (Zuccotti et al., 2015). Accordingly, commensal intestinal microbes interact through complex community dynamics to collaboratively promote microbiota euboisis (Ha et al., 2014; Coyte and Rakoff-Nahoum, 2019). For instance, *Lactobacilli* aids mucus binding of *Bifidobacteria* (Ouwehand et al., 2000), enabling greater functionality than when alone. Moreover, a major means of microbial interaction is via metabolic cross-feeding, in which certain intestinal bacteria feed off the metabolic products, such as SCFAs (Hirmas et al., 2022; Culp and Goodman, 2023), of other microbes, resulting in a complex network of producers and consumers cooperating to support microbiota functionality. As such, the use of multi-strain microbial cocktails supports the natural microbial interplay of the human microbiome, thus likely enhancing LBP efficacy. Therefore, future research should examine the use of multi-strain bacterial consortiums in allergy therapeutics, compared to traditional single-strain interventions, and determine which strains function best synergistically to provide optimal pro-tolerogenic effects.

Furthermore, although it is well established that the intestinal microbiota plays a crucial role in immune development and allergy susceptibility, what constitutes a microbiota composition that promotes and maintains immunotolerance to allergens is still unclear. Greater research to further establish this and the underlying immunological mechanisms, i.e. IgA-dependent pathways, is of significant value to inform microbiota-targeted interventions for reducing allergy risk.

Lastly, the prophylactic vs. therapeutic potential of LBPs in allergy remains unclear. While the majority of research thus far assesses the role of LBPs in the prevention of allergy development, greater research should examine whether LBPs can be used therapeutically to promote immunotolerance to pre-sensitized allergens in existing allergic disease. Although sparse, a small number of studies thus far have assessed the therapeutic role of LBPs on existing allergy; Supplementation of *Lactobacillus paracasei* and *Lactobacillus fermentum* both combined and alone were found to decrease asthma severity scores and serum IgE levels, and improve peak excitatory flow rates (PEFR) in asthmatic children aged 6–18 years old (Huang et al., 2018). Similarly, 8 week supplementation of *Lactobacillus gasseri* in children aged 6–12 years old, significantly reduced clinical symptoms of asthma, improved pulmonary function and PEFR and reduced allergen-stimulated production of inflammatory cytokines including IL-12, IL-13 and TNF-α (Chen et al., 2010). However, it is important to note the limited follow-up periods of these studies. In fact, RCTs with longer follow-up periods have reported negative results, including *Lactobacillus salivarius* at 4 month follow-up (Lee S-C. et al., 2013), *Lactobacillus rhamnosus GG* at 6 month follow-up (Rose et al., 2010), and *Lactobacillus bulgaricus* and *Streptococcus thermophilus* at 12-month follow-up (Giovannini et al., 2007), all demonstrating no benefits on allergy severity. This suggests the therapeutic effects of LBP supplementation in managing existing allergic disease may be temporary, and not persist beyond the supplementation period. Long-term daily supplementation may provide longer lasting benefits for the management of allergy symptoms. Consequently, greater research is necessary to decipher the therapeutic, compared to prophylactic, role of LBP interventions in promoting immunotolerance and managing existing allergic disease.

### Conclusion

Overall, LBPs demonstrate powerful immunomodulatory effects during the early-life critical window and have exciting potential to be harnessed as a therapeutic tool for childhood allergic disease. Both animal and human data suggest that LBPs may act through several immunological and physiological mechanisms to influence allergic immune response, including but not limited to, TLR-induced regulation of Th1/Th2 balance, the production of microbial-derived SCFAs and resulting immunomodulation via GPR41/GPR43 activation and HDAC inhibition, and strengthening of epithelial barrier integrity. However, despite highly promising animal data, findings from human intervention trials are mixed and inconsistent. The efficacy of LBPs in allergy protection is not straightforward and reflects a complex interplay of multiple factors including microbial strain, duration/timing, population genetics, infant risk status, maternal and lifestyle factors, and allergy phenotype. Despite this, LBPs hold exciting potential for both the prevention and treatment of allergic disease across the globe. Future research, particularly regarding optimal maternal/infant supplement regimes, and the efficacy of multi-strain consortiums and synbiotics, is necessary to translate findings into clinical applications, representing a fruitful and promising field of investigation.

## Author contributions

IT: Conceptualization, Writing – original draft, Writing – review & editing. BF: Funding acquisition, Supervision, Writing – review & editing.
